# Supplementary material for: Kill two birds with one stone: making multi-transgenic pre-diabetes mouse models through insulin resistance and pancreatic apoptosis pathogenesis
Source: PeerJ. 2018 Apr 17;6:e4542. doi: 10.7717/peerj.4542 (PMC5909684; doi:10.7717/peerj.4542)
Supplement: Table S1 [file peerj-06-4542-s001.docx]

| Primer | Sequence (5’-3’) | Anealing temp(℃) | Product (bp) | Use | Reference |
| --- | --- | --- | --- | --- | --- |
| 11β-HSD1 F | GCTCCCTTTCCCCCTTAACC | 60 | 513 | Triple-transgenic mice | [1] |
| 11β-HSD1 R | AGGCCAAGAAGATCCCCAGA |  |  |  |  |
| CHOP-3 F | AGGGAAATGATCCAGAAAGTGC | 57 | 506 | Triple-transgenic mice | [1] |
| CHOP-3 R | GGACGCAGGGTCAAGAGTAGTG |  |  |  |  |
| hIAPP-3 F | GAAACGGAAACAGAGTGG | 57 | 445 | Triple-transgenic mice | [1] |
| hIAPP-3 R | GTTGCTGGAATGAACTAAAA |  |  |  |  |
| CHOP-1 F | ATCTCGGCAGGAGGACG | 60 | 358 | CHOP-transgenic mice | n/a |
| CHOP-1 R | GCTTTGGGATGTGCGTGT |  |  |  |  |
| hIAPP-2 F | CTTCCTCAGCTCCTTCCA | 58 | 910 | Double-transgenic mice | n/a |
| hIAPP-2 R | CTCCGCTCCATCGTTCA |  |  |  |  |

**Reference**

1. Kong S, Jinxue R, Xin L, et al. Multi-transgenic minipig models exhibiting potential for hepatic insulin resistance and pancreatic apoptosis[J]. Molecular Medicine Reports, 2016, 13(1):669-680.
