# Supplementary material for: Kill two birds with one stone: making multi-transgenic pre-diabetes mouse models through insulin resistance and pancreatic apoptosis pathogenesis
Source: PeerJ. 2018 Apr 17;6:e4542. doi: 10.7717/peerj.4542 (PMC5909684; doi:10.7717/peerj.4542)
Supplement: Supplemental Information 1 [file peerj-06-4542-s002.pdf]

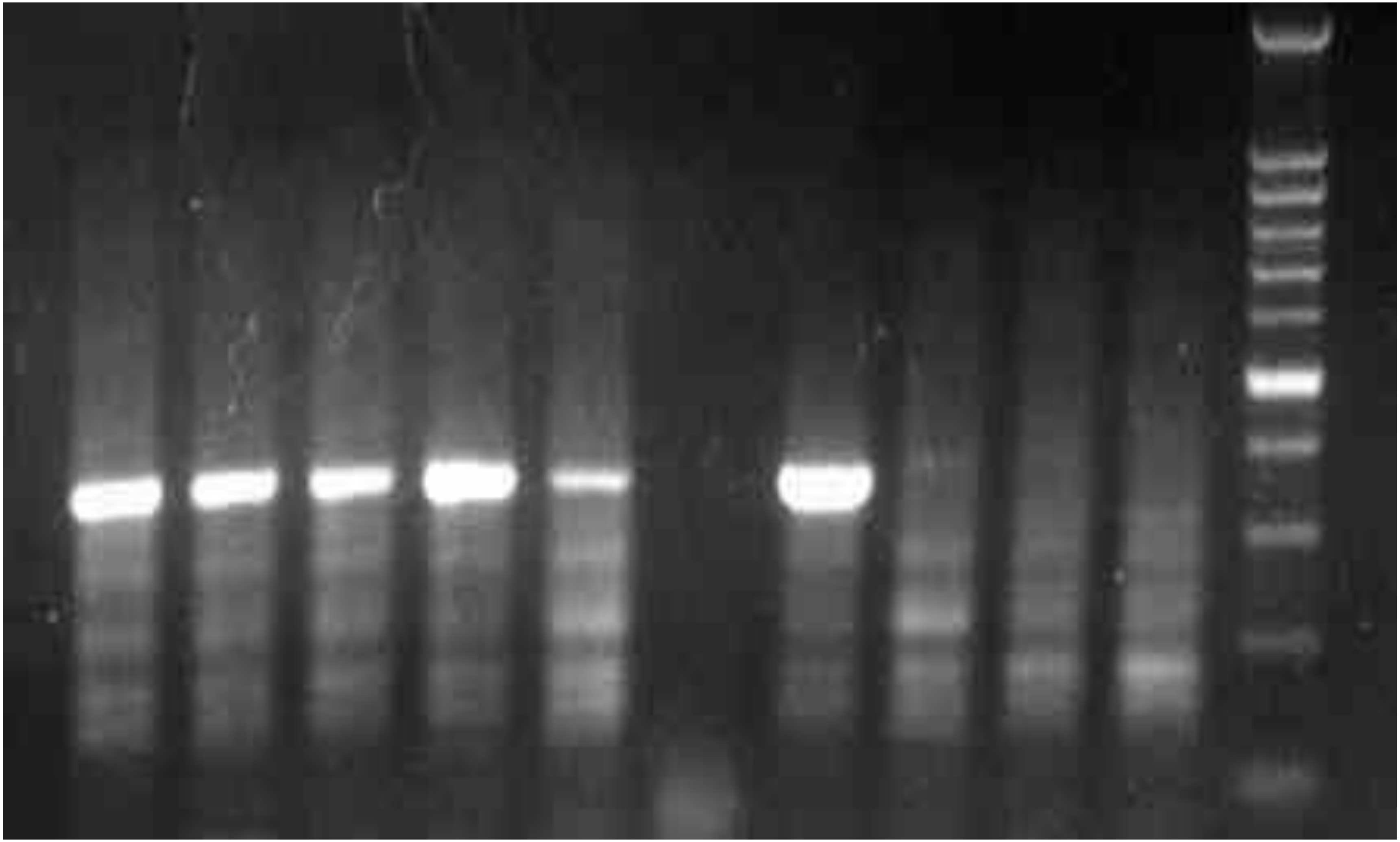

Fig. S1 Positive PCR of the CHOP (358 bp) transgenic mice.

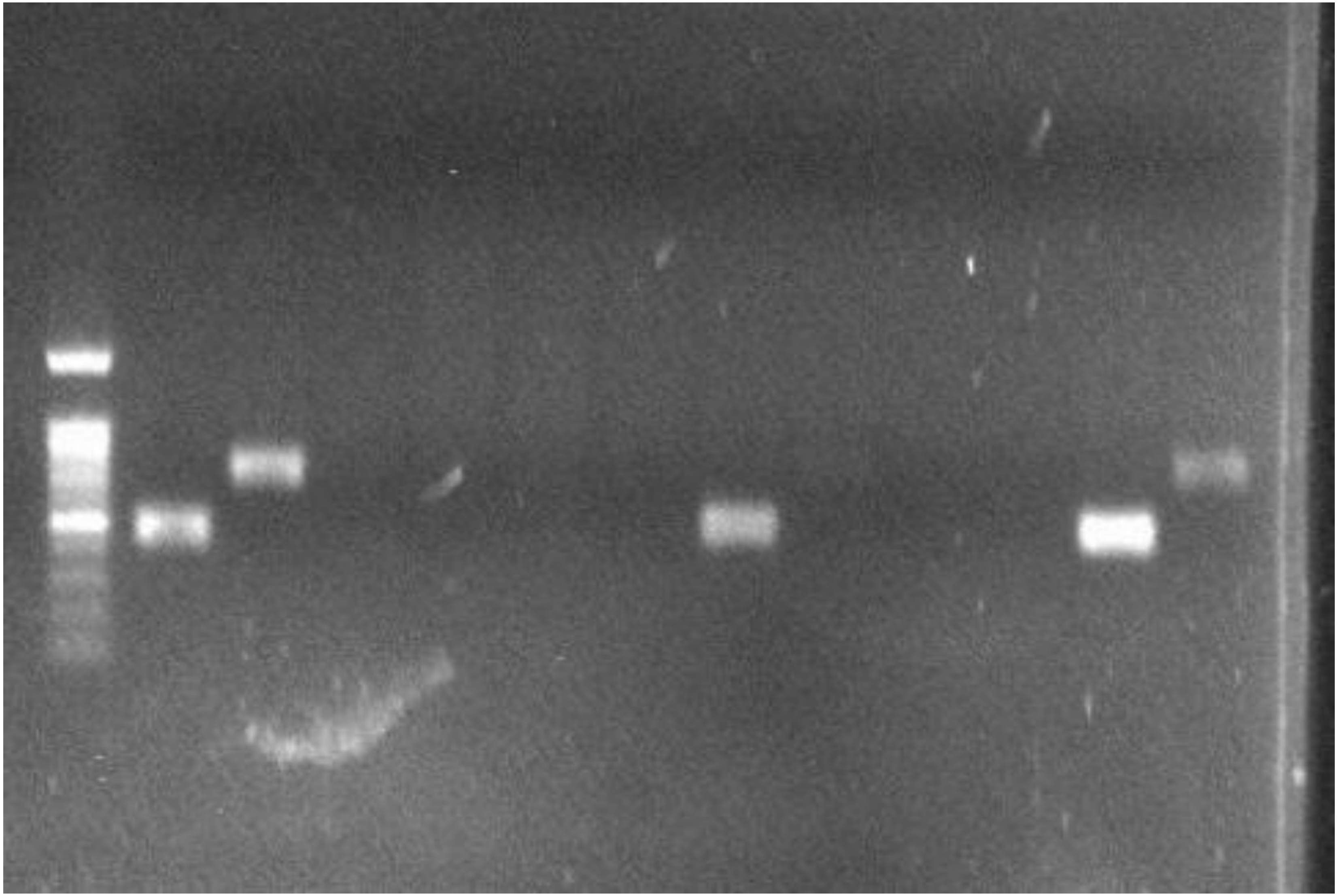

Fig. S2 Positive PCR of the CHOP (506 bp) and hIAPP (910 bp) transgene mice.

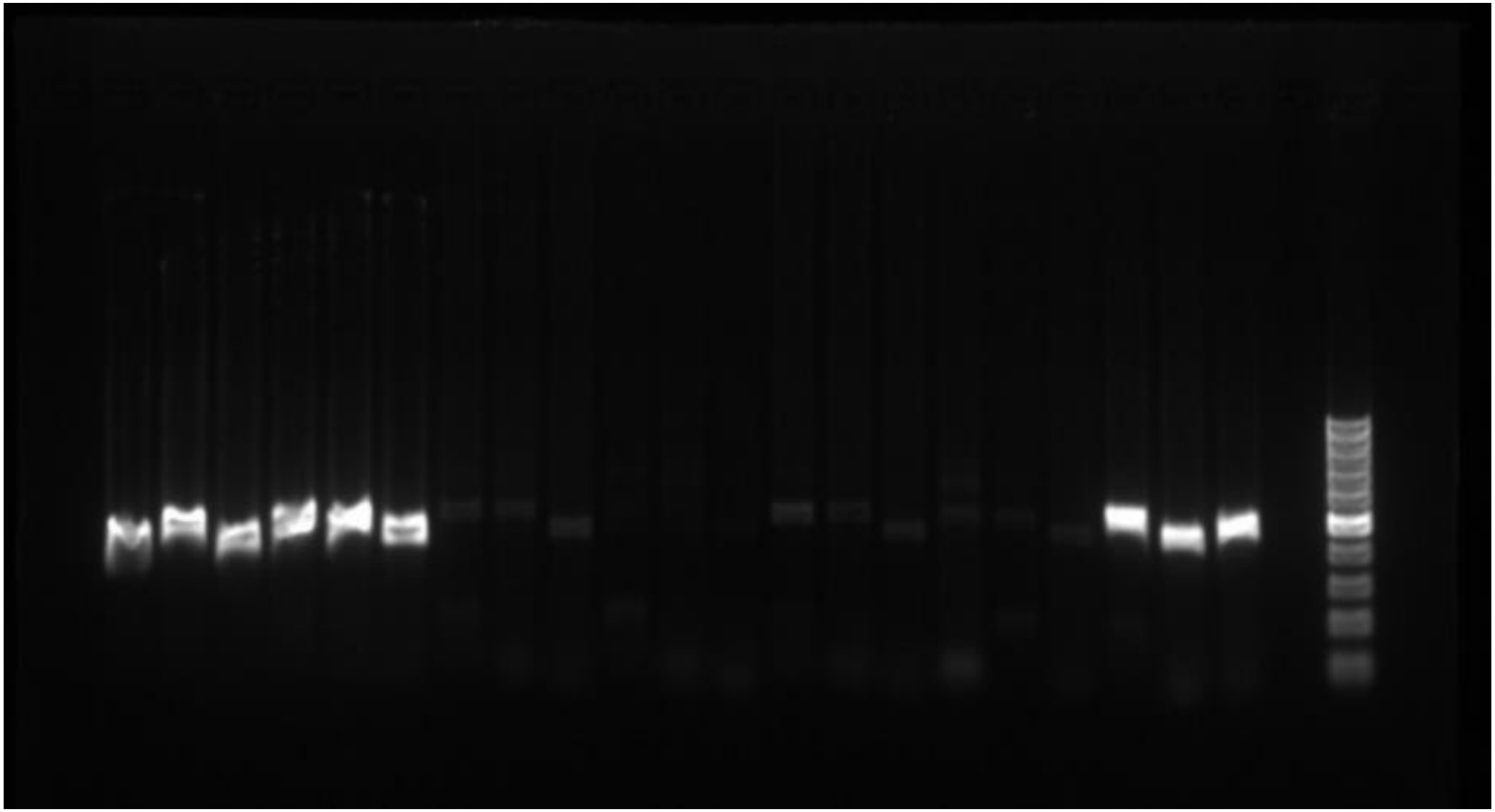

Fig. S3 Positive PCR of the11β-HSD1 (513 bp), CHOP (506 bp) and hIAPP (445 bp) transgenic mice
